# Is the Mannheim Multicomponent Stress Test a viable alternative to the Trier Social Stress Test?

**DOI:** 10.1016/j.cpnec.2024.100275

**Published:** 2024-11-07

**Authors:** Daniel S. Kashi, Marianne Hunter, Jason P. Edwards, Harry Bell, Megan Robinson, Neil P. Walsh

**Affiliations:** Faculty of Science, Liverpool John Moores University, Liverpool, UK

**Keywords:** Acute stress, Salivary cortisol, TSST, HPA-Axis, Social evaluative threat, Stress reactivity

## Abstract

**Background:**

The Trier Social Stress Test (TSST) is a widely used laboratory protocol to study acute stress reactivity, a hallmark of which is a meaningful increase in saliva cortisol (>2.5 nmol/L) in most individuals, reflecting hypothalamic-pituitary-adrenal (HPA) axis activation. The Mannheim Multicomponent Stress Test (MMST) has potential as a low staff burden alternative to the TSST, with one study showing statistically significant increases in subjective stress, heart rate and saliva cortisol; however, uncertainty remains about the meaningfulness of these psychobiological responses.

**Objective:**

To assess whether the MMST is a viable alternative to the TSST.

**Methods:**

Using a between subjects design, 31 healthy adults were randomised to the standard TSST or the MMST using stratified block randomisation accounting for sex and trait anxiety. The standard TSST consisted of an anticipation phase, followed by a free speech and mental arithmetic task performed in front of a panel of trained actors. The MMST consisted of a computer based Paced Auditory Serial Addition Task (cognitive stressor) with additional motivational, emotional and acoustic stressors in the presence of one unresponsive observer.

**Results:**

Group × time interactions showed that the MMST induced smaller psychobiological responses compared with the TSST (mixed model ANCOVA, *P* < 0.05). Post-hoc analyses revealed that the MMST induced a significant yet smaller state anxiety response (score range 20–80, MMST: 47 ± 12 *vs.* TSST: 57 ± 9; *P* < 0.01, Cohens *d* = 0.9) and peak heart rate response (MMST: 98 ± 17 *vs.* TSST: 110 ± 21 bpm; *P* < 0.05, Cohens *d* = 0.6) compared with the TSST. Despite observing stereotypical neuroendocrine responses to the TSST, the MMST did not increase saliva α-amylase or cortisol (Δ saliva cortisol, 0.1 ± 1.1 *vs.* TSST: 10.3 ± 12.8 nmol/L; between group difference *P* < 0.01, Cohens *d* = 1.1). Moreover, meaningful increases in saliva cortisol (>2.5 nmol/L) were observed in 80% of participants after the TSST but in no participant after the MMST.

**Conclusion:**

The Mannheim Multicomponent Stress Test increased state anxiety and heart rate but not saliva cortisol. As such, the present results do not support the utility of the Mannheim Multicomponent Stress Test as a viable alternative to The Trier Social Stress Test.

## Highlights

• The MMST may offer a low staff burden alternative to the TSST.
• MMST successfully increased state anxiety and heart rate, but not saliva cortisol.
• TSST elicited robust increases in state anxiety, heart rate and saliva cortisol.
• The MMST is not a viable alternative to the TSST to elicit HPA-axis activation.

## 1 Introduction

Findings from laboratory studies investigating psychobiological responses to acute stress have improved understanding of the long-term adverse health and behavioural outcomes associated with various psychopathologies including, depression, anxiety disorders, eating disorders and substance dependencies (e.g., smoking, alcohol and recreational drug use) along with exhaustion, chronic fatigue syndrome and irritable bowel syndrome [[1], [2], [3]]. Indeed, dysregulation of the acute stress response (i.e., blunted or exaggerated reactivity) has been associated with cognitive decline and cardiovascular disease mortality [[4], [5]] highlighting the translational value of employing standardised laboratory stress tests that elicit meaningful psychobiological responses [1].

The Trier Social Stress Test (TSST) [6] is a commonly used protocol to study acute stress reactivity in the laboratory, typically eliciting increases in salivary levels of α-amylase, a surrogate marker of sympathetic-adrenal-medullary (SAM) axis activation, and cortisol, the stress hormone output from the hypothalamic-pituitary-adrenal (HPA) axis [7,8]. Fundamental to the TSST eliciting meaningful HPA reactivity is the uncontrollable and unpredictable experience of social evaluative threat, created by challenging the participant to deliver a free speech and complete an unexpected mental arithmetic test in front of an unresponsive panel of between two and four trained actors. Besides the significant staff resource burden, for example one study reported training 10 actors as TSST panel members [9], implementation of the social evaluation element accounts for significant variability in psychobiological responses to the TSST between laboratories, e.g., the number of panel members influences HPA reactivity to the TSST [10].

Low staff burden alternatives to the TSST include the Social Evaluative Cold Pressor Task [11], the Maastricht Acute Stress Test [12] and the less-studied Mannheim Multicomponent Stress Test (MMST) [13]. The MMST incorporates a combination of cognitive, motivational, emotional and acoustic stressors. During the test the participant completes the computer based paced auditory serial addition task (PASAT-C), which reliably perturbs cardiovascular responses [14,15], in the presence of one observer and is informed that incorrect answers result in reduced monetary compensation. Simultaneously, the participant is exposed to emotionally evocative images and white noise via headphones. Besides being less labour-intensive than the TSST, from a standardisation perspective the MMST is likely less susceptible to variable psychobiological responses attributable to the social evaluation component in the TSST. To date, only one study has investigated the psychobiological responses to the MMST showing promising elevations in subjective stress and heart rate and modest saliva cortisol reactivity [13]. Meaningful increases in saliva cortisol (>2.5 nmol/l) were observed in only half of all participants after the MMST yet the TSST typically elicits robust HPA reactivity with meaningful increases in saliva cortisol in >70 % of participants [6].

With this information in mind, in a randomised between groups design, we assessed the utility of the MMST to elicit meaningful psychobiological responses compared with the TSST.

## 2 Methods

This study received local ethical approval (Liverpool John Moores University Research Ethics Committee: 20/SPS/043) and protocols were conducted in accordance with the Declaration of Helsinki (2013).

### 2.1 Participant recruitment and exclusion

Healthy adults were recruited from the local population and eligibility was assessed using a self-report health screening questionnaire. All participants were healthy adults aged 18–35 years, non-smokers, free from any known immune, cardiovascular, or metabolic diseases, and were not taking prescription medication, except for females prescribed oral combined contraception (OCC). Use of the OCC pill and self-reported stable menstrual cycle were inclusion criteria for females.

### 2.2 Familiarisation

Participants visited the laboratory for a familiarisation visit, where a member of the research team showed participants the laboratory suite, questionnaires and the saliva sampling protocol used during the subsequent trials. Considering the known influences of trait anxiety recent life stress and sleep quality on psychobiological responses to acute stress [[16], [17], [18]], at the familiarisation visit participants also completed the State-Trait anxiety inventory (STAI-T) [19] perceived stress scale [20] and the Pittsburgh Sleep Quality Index [21]. During the familiarisation visit, participants were not explicitly told what the experimental trials would entail, only that they would complete a challenging mental performance task. After completing the study, participants attended a study debriefing where they were fully informed about the primary study aim (i.e., to induce an acute stress response rather than to assess mental performance) and the reward they would receive for their participation (£25).

### 2.3 Standardisation and controls for experimental trials

To minimize the impact of circadian variations in saliva cortisol and alpha amylase, experimental trials took place between 1200 and 1700 h. In addition, participants were asked to refrain from unfamiliar or exhaustive exercise and alcohol consumption in the 24 h prior to the trials, to avoid caffeine on the morning of the experimental trials and avoid food intake and brushing their teeth in the 2 h prior to the start of the trial. Experimental trials were performed during the active pill taking phase in OCC using females (*n* = 4, Table 1). All other females not using OCC completed experimental trials during the follicular phase i.e., days 1–8 following self-report onset of menses.

**Table 1:** Baseline descriptive information for study participants.

	All	MMST	TSST
	*N* = 31	N = 16	*N* = 15
*Demographic*			
Age, years	23 ± 3	23 ± 4	23 ± 3
BMI (kg/m^2^)	24 ± 3	23 ± 3	25 ± 3
Sex, Male [*N* (%)]	18 (58)	9 (56)	9 (60)
Female OCC user	4 of 13	2 of 7	2 of 6

Trait anxiety rowhead			
Score (20–80)	37 ± 9	37 ± 9	37 ± 9

*Perceived stress in the month before study enrolment*			
Score (0–40)	13 ± 6	13 ± 5	14 ± 6

*Sleep quality in the month before study enrolment*			
Score (0–21)	2 ± 1	2 ± 1	2 ± 1

Values presented as mean ± SD unless otherwise stated. MMST = The Mannheim multicomponent stress test; TSST = The Trier social stress test; BMI = Body mass index; OCC = Oral combined contraception.

### 2.4 Study design and procedures

The study adopted a between subjects design to compare psychobiological responses in participants randomly assigned to either the MMST or the traditional TSST protocol. Stratified block randomisation was used to evenly distribute participants to the two stress tests based upon sex (male or female) and trait anxiety (low STAI-T score <40 or high STAI-T score ≥40) [19], as these are widely recognised to influence psychobiological responses to acute stress [22].

### 2.5 Acute stress test protocols: MMST and the Trier social stress test

Following a 30 min period of seated rest in a reception area, participants were exposed to either the MMST or the TSST. The TSST consists of a preparation phase (5 min), followed by a mock job interview and a mental arithmetic task (5 min each) in front of two observers (1 male and 1 female, both unknown to the participant) and a video recording device, as described [6]. For the MMST protocol each participant was escorted to a stress induction room. The MMST applies stressors of different modalities (cognitive, acoustic, emotional and motivational) as described [13]. As a cognitive stressor, the PASAT-C was performed which requires the participant to sum two sequentially presented numbers in the range from 0 to 20 by clicking on the correct answer using a computer mouse. After providing each sum, the participant had to ignore the sum and add the following number to the number most recently presented. The PASAT-C consists of two levels, each lasting 2 min, in this MMST protocol the time latency between the numbers was 4 and 2 s across levels 1 and 2. As an emotional stressor, 44 negative related pictures displaying violence, hurt humans/animals and suffering retrieved from the International affective picture system were presented for 5 s on a larger screen positioned slightly above the PASAT-C in the participant’s field of vision. Five negative images were followed by one positive picture (e.g., idyllic landscapes and smiling babies) for 3 s to avoid habituation. To direct a participant’s attention to the images, they were asked to verbally detect the images presented twice during an initial 1 min phase before the start of the PASAT-C. White noise presented over headphones was constantly applied during the stress induction to act as an acoustic stressor. The intensity of the white noise was increased from 85 to 93 dB between levels 1 and 2 to avoid habituation. To add a motivational stressor, an element of deception was used whereby the participant was told that they would lose their £25 reimbursement if their performance did not improve between PASAT levels 1 and 2 whereas, in reality, the participation reward was £25. Throughout the duration of the MMST, an evaluator of the opposite sex, unknown to the participant and dressed in a white laboratory coat was present in the stress induction room. The evaluator sat within eye line of the participant and was instructed to watch the participant, read from a standardised script, and remain neutral and emotionless throughout the duration of the task. Once the test was completed, the participant was escorted out of the stress induction room and remained seated in a reception area during a recovery period lasting 1 h.

### 2.6 Study measures

#### 2.6.1 Anxiety and heart rate

Anxiety was measured using the state aspect of the state anxiety inventory (STAI) [19]. The STAI questionnaire contains 20 items, and each item is rated on a 4-point Likert scale (range from ‘1’ = not at all to ‘4’ = very much). The total scores of this measure are obtained by summing the values assigned to each item and range from a minimum of 20 to a maximum of 80, with higher score indicating more severe anxiety symptoms. The STAI was administered to participants at −30 min (baseline), −5 min (pre-test), immediately post and 60 min post stress tests. Heart rate was monitored continuously during experimental trials (Polar H10, Polar Electro, Kempele, Finland).

#### 2.6.2 Saliva cortisol and α-amylase assessment

Saliva samples were collected using a Salivette device (Sarstedt, Numbrecht, Germany) and participants were asked to chew the swab for 1 min. Saliva samples were obtained at −30 min (baseline), −5 min (pre), 0 (post) and +10, +20, +30- and +60-min post stress tests. Saliva was extracted from cotton swabs by centrifugation per the manufacturer’s instructions and frozen in multiple aliquots at −80 °C until thawing for biochemical analysis. Saliva samples were analysed for α-amylase using a quantitative enzyme kinetic method, as described [23] and for free cortisol by enzyme linked immunosorbent assay (Salimetrics, State College, PA). The mean intra-assay coefficient of variation was 4.2% and 5.3% for saliva α-amylase and cortisol, respectively.

#### 2.6.3 Statistical analysis

All analyses were conducted using SPSS 29.0 (IBM, Armonk, NY, USA) with statistical significance set at *P* < 0.05. All data were checked for normality and sphericity and where sphericity was violated Greenhouse Geisser correction was applied to the degrees of freedom. Participant demographic data are presented as mean ± SD for continuous variables or absolute numbers and percentages for categorical variables; comparisons were made using independent t-tests and chi-square analysis, where appropriate (Table 1). Sample size was estimated at thirty participants to detect a significant difference in the saliva cortisol response between the MMST and TSST (Δ, pre-test *vs*. peak post-test), with alpha set at 0.05, power at 0.8 and Cohen’s *d* effect size of 0.95. The effect size for this estimation was calculated using in-house pilot data for the MMST compared with published data from a re-analysis of five separate studies assessing the saliva cortisol response to the TSST [24]. In response to stress, saliva α-amylase reaches its peak and recovers faster compared with saliva cortisol [25]. As such, ‘Peak’ values for saliva α-amylase were established using immediately post-test concentrations and ‘Peak’ values for saliva cortisol were recorded as the highest value observed from ‘Post’ to +60 min in the post-test period, as described [24,26]. Independent T-tests were conducted to compare the change (Δ) in saliva cortisol and α-amylase between the MMST and TSST (Δ saliva cortisol, pre-test *vs*. peak post-test and Δ saliva α-amylase, pre-test *vs*. immediately post-test). With generalisability in mind, we also report saliva cortisol responders as those exhibiting a >2.5 nmol/L increase from pre-test to peak post-test, as is widely adopted [8,27]. As a meaningful increase in saliva α-amylase is not clearly defined, a pilot investigation was conducted to establish the typical day-to-day biological variation (Supplementary Table 1); a meaningful responder to the stress test was determined when the delta change in saliva α-amylase was greater than the intraindividual coefficient of variation (CV_I_, 25 %), as described [28,29]. We also compared all psychobiological responses to the MMST and TSST using mixed-model analysis of covariance (ANCOVA), with ‘Baseline’ as the covariate, with post hoc Bonferroni pair-wise comparisons. In addition, we assessed the efficacy of the MMST and TSST to elicit high state anxiety by comparing the number of participants with a post-test state anxiety score ≥40 [19]. The magnitude of effect for T-test comparisons was reported using Cohen's d, where 0.2, 0.5, and 0.8 represent small, medium, and large effects, respectively [30]. For ANOVA, the effect sizes are reported as the partial η^2^ value, where 0.01, 0.06, and 0.14 represent small, medium, and large effects, respectively [30].

## 3 Results

### 3.1 Participant flow and baseline descriptives

Thirty-seven heathy adults provided written informed consent and were randomly assigned to complete either the MMST or TSST; participant flow, exclusion and drop out before analysis is summarised in Supplementary Fig. 1. There were no significant differences between participants randomly assigned to the MMST or TSST for descriptive information, including: basic demographics, trait anxiety, recent life stress, sleep quality and OCC use among females (Table 1).

### 3.2 The MMST stimulates psychobiological responses but to a lesser extent than the TSST

**Fig. 1:**
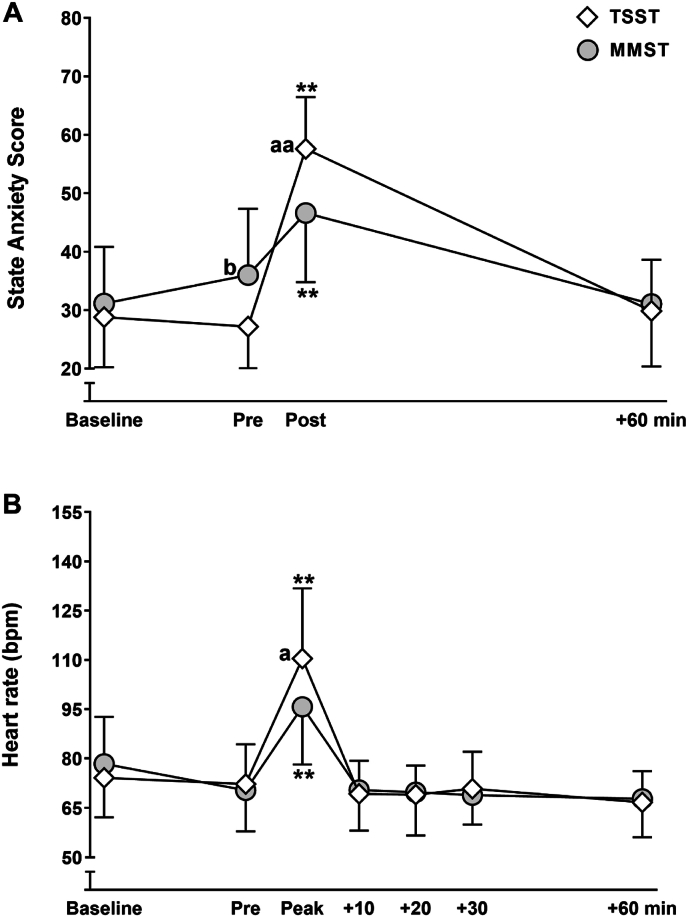
State anxiety (A) and heart rate (B) responses to the Mannheim Multicomponent stress test (MMST, n = 16) and the Trier Social Stress Test (TSST, n = 15). Values are presented as mean ± SD. ∗∗ greater than ‘Pre’, *P* < 0.01. ^a^ greater than MMST, *P* < 0.05, ^aa^ greater than MMST, *P* < 0.01. ^b^ greater than TSST, *P* < 0.05.

A similar number of participants assigned to the MMST and TSST reported high state anxiety 5 min before the stress test exposure; 2 of 16 reported a score ≥40 before the MMST and 1 of 15 before the TSST. Mixed model ANCOVA, showed that both the TSST and MMST elicited significant increases in state anxiety and heart rate (*P* < 0.01; Fig. 1A and B). However, an interaction indicated that state anxiety and heart rate were increased to a greater extent by the TSST compared with the MMST (state anxiety: F(2, 56) = 7.0, *P* < 0.01, η^2^ = 0.30, heart rate: F(2, 48) = 3.7, *P* < 0.05, η^2^ = 0.12). Notwithstanding, the MMST successfully evoked high state anxiety in 11 of 16 participants (*vs*. all 15 participants who completed the TSST), and all but 2 participants mounted meaningful heart rate responses during the MMST that exceeded the typical day-to-day variation (Supplementary Table 1).

**Fig. 2:**
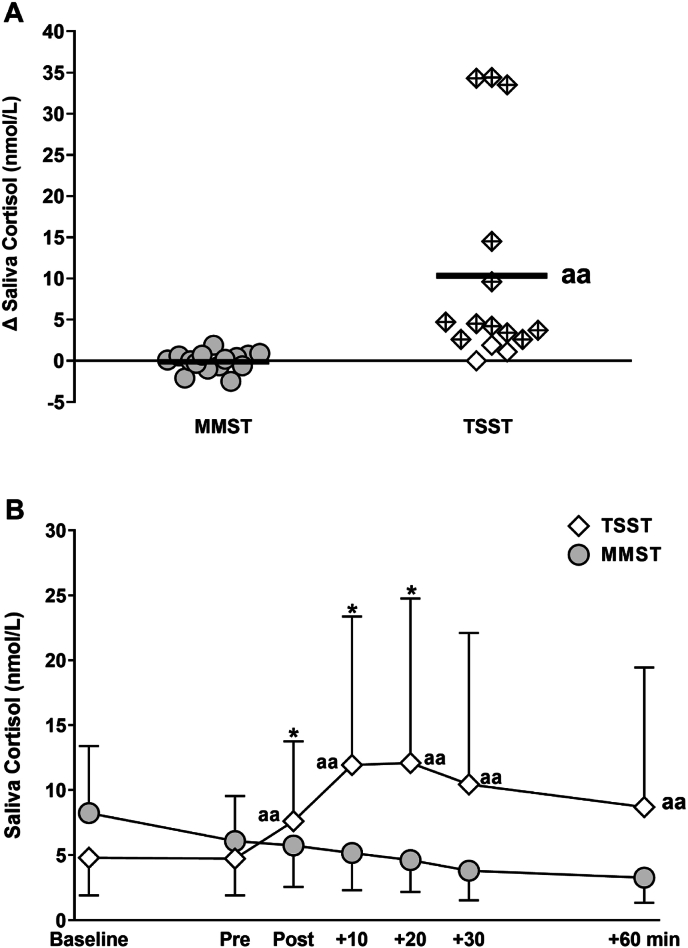
Saliva cortisol response to the Mannheim Multicomponent stress test (MMST, n = 16) and the Trier Social Stress Test (TSST, n = 15). Values are presented as the individual change from pre-test to peak posttest (panel A) and as mean ± SD (panel B). In panel A, the unbroken horizontal line shows the mean change and shapes with a cross represent meaningful responders, defined as an increase in saliva cortisol >2.5 nmol/L, as described [8]. ∗ greater than ‘Pre, *P* < 0.05. ^aa^ greater than MMST, *P* < 0.01.

Despite successfully increasing state anxiety and heart rate, the MMST did not elicit statistically significant or meaningful increases in saliva cortisol (*P* > 0.05, Cohens *d* = 0.03, Fig. 2). In contrast, we observed stereotypical increases in saliva cortisol from pre to peak post-test in response to the TSST (Δ saliva cortisol *vs.* MMST, *P <* 0.01, Cohens *d* = 1.1, Fig. 2A). Indeed, in response to the TSST, meaningful saliva cortisol responses that exceeded the typical day-to-day variation (Supplementary Table 1) were observed in 14 of 15 participants and in 12 of 15 when applying the widely adopted >2.5 nmol/L increase in saliva cortisol from pre to peak post TSST. After accounting for differences at baseline, saliva cortisol responses were absent following the MMST, whereas significant increases were observed in response to the TSST (mixed model ANCOVA group × time interaction: F(2, 48) = 5.6, P < 0.01, η^2^ = 0.12, Fig. 2B).

**Fig. 3:**
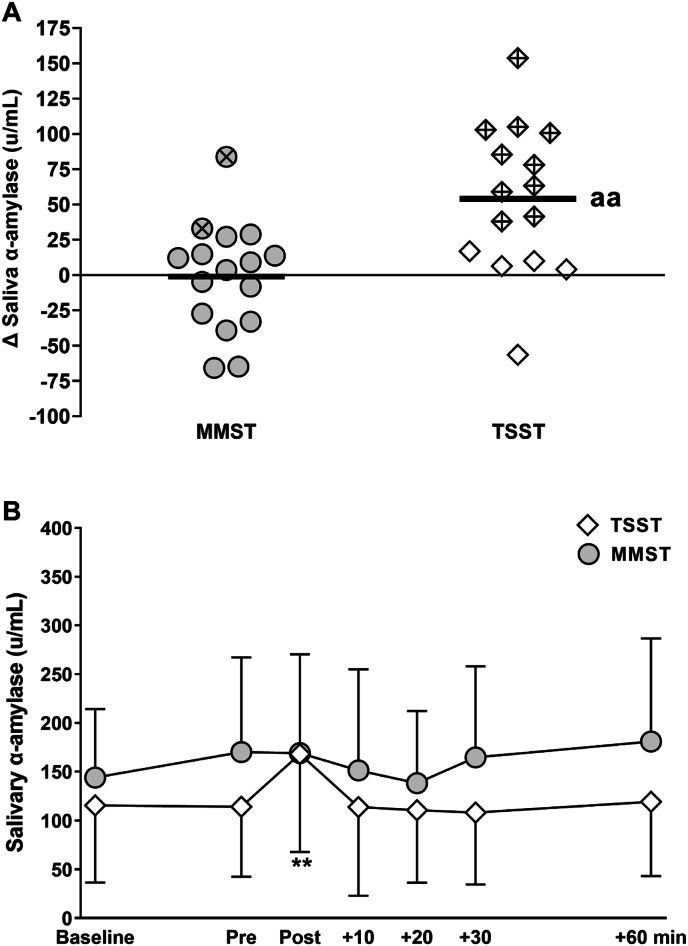
Saliva α-amylase response to the Mannheim Multicomponent Stress Test (MMST, n = 16) and the Trier Social Stress Test (TSST, n = 15). Values are presented as the individual change from pre-test to immediately posttest (panel A) and as mean ± SD (panel B). In panel A, the unbroken horizontal line shows the mean change and shapes with a cross represent meaningful responders where saliva α-amylase increased >25%, exceeding the typical day-to-day biological variation shown in a separate pilot investigation (Supplementary Table 1). ∗∗ greater than ‘Pre, *P* < 0.01. ^aa^ greater than MMST, *P* < 0.01.

In terms of SAM-axis activation, the MMST did not evoke statistically significant increases in saliva α-amylase (*P* > 0.05, Cohens *d =* 0.01, Fig. 3). By comparison, the TSST caused significant increases in saliva α-amylase from pre to immediately post-test (Δ saliva α-amylase *vs.* MMST, *P <* 0.01, Cohens *d* = 1.2, Fig. 3A). In addition, meaningful saliva α-amylase responses, that exceeded the typical day-to-day variation (Supplementary Table 1), were observed in only 2 of 16 participants after the MMST *vs*. 10 of 15 participants who completed the TSST. After accounting for differences at baseline, the TSST exhibited a robust increase in saliva α-amylase, whereas responses were absent following the MMST (mixed model ANCOVA group × time interaction: F(3, 95) = 2.9, *P* = 0.03, η^2^ = 0.10, Fig. 3B).

## 4 Discussion

The Trier Social Stress Test (TSST) is a commonly used protocol to study acute stress responses in the laboratory, characterised by robust HPA reactivity with meaningful increases in saliva cortisol typically in >70% of participants [6]. Preliminary findings, albeit from only one study, show that the MMST induces more subtle saliva cortisol responses, meaningful in approximately half of all participants [13]. Mindful of the challenges standardising the social evaluative component of the TSST and the associated variability in HPA-axis reactivity reported between laboratories [10], here in a between groups design, we directly compared acute stress responses to the MMST and TSST. Stratified block randomisation was used to evenly distribute participants to the two stress tests based upon sex and trait anxiety, factors widely recognised to influence psychobiological responses to acute stress [22,5]. Success of the stratified block randomisation was evidenced by a similar number of participants reporting high state anxiety 5 min before stress test exposure in MMST and TSST. Furthermore, other factors considered to influence stress reactivity including BMI, recent life stress and sleep quality were also comparable between groups (Table 1, [16,22,5]). Consistent with Reinhardt and colleagues, we show that the MMST is effective in eliciting significant increases in subjective and cardiovascular measures of stress reactivity, here indexed by state anxiety score and heart rate, respectively. However, we did not observe significant or meaningful increases in either saliva α-amylase or cortisol after the MMST. Conversely, among a matched sample, we were able to evoke stereotypical increases in saliva cortisol using the traditional TSST protocol, with 80 % of responses considered meaningful (>2.5 nmol/L, [6]).

Whilst we cannot fully account for the lack of HPA-axis reactivity to the MMST in the present study, the divergent findings compared to Reinhardt et al. might be explained by between-study differences in participant characteristics (e.g., trait anxiety), psychosocial factors (e.g., recent life stress), sleep quality and the menstrual status of females at the time of the stressor [22]. Reinhardt et al. did not present information on trait anxiety, recent stress, or sleep quality, as such we cannot rule out that the modest increase in saliva cortisol in response to the MMST in their study (∼3 nmol/L on average) might relate to differences in these factors between the studies. Whilst the proportion of females in the present study (42%) was comparable to Reinhardt et al. (50%), we scheduled experimental trials in the follicular phase and included OCC using females, whereas they scheduled trials during the luteal phase and excluded OCC users. Cortisol reactivity during the luteal phase is reportedly robust and comparable to males and greater than cortisol reactivity in the follicular phase and in OCC using females [31]. Notwithstanding, it’s noteworthy that if we restrict our observations to males alone, unlike the TSST, the MMST did not induce significant or meaningful saliva cortisol reactivity (Δ saliva cortisol: MMST = *-*0.3 ± 0.9 *vs.* TSST = 14.0 ± 14.4 nmol/L). Compared to the TSST, selecting more subtle stress paradigms that place less emphasis on social evaluation, like the MMST, risks cortisol non-responsivity, and the efficacy to evoke meaningful saliva cortisol responses is likely influenced to a greater extent by participant characteristics, psychosocial and lifestyle factors.

These results show that the MMST increased state anxiety and heart rate but not saliva cortisol or α-amylase. By contrast, in participants matched for sex and trait anxiety, we observed stereotypical and robust psychobiological responses to the TSST; notably, meaningful saliva cortisol and α-amylase responses were observed in the majority. Despite the MMST offering advantages in terms of standardisation and low resource burden, the TSST remains a more potent inducer of psychobiological responses. In conclusion, the present results do not support the utility of the Mannheim Multicomponent Stress Test as a viable alternative to The Trier Social Stress Test.

## CRediT authorship contribution statement

**Daniel S. Kashi:** Writing – original draft, Supervision, Project administration, Methodology, Investigation, Formal analysis, Data curation, Conceptualization. **Marianne Hunter:** Project administration, Methodology, Conceptualization. **Jason P. Edwards:** Writing – review & editing, Methodology, Formal analysis. **Harry Bell:** Project administration, Methodology, Investigation. **Megan Robinson:** Methodology, Formal analysis. **Neil P. Walsh:** Writing – review & editing, Supervision, Conceptualization.

## Clinical trial registry number

NCT06066320 https://clinicaltrials.gov/study/NCT06066320.

## Role of the funding source

The study received no direct financial support, but the first author’s post-doctoral position was funded by Danone Global Research & Innovation Center

## Declaration of competing interest

The authors declare the following financial interests/personal relationships which may be considered as potential competing interests: Dr. Daniel Kashi reports financial support was provided by Danone Global Research & Innovation Center. If there are other authors, they declare that they have no known competing financial interests or personal relationships that could have appeared to influence the work reported in this paper.
