# Supplementary material for: Is the Mannheim Multicomponent Stress Test a viable alternative to the Trier Social Stress Test?
Source: Compr Psychoneuroendocrinol. 2024 Nov 7;20:100275. doi: 10.1016/j.cpnec.2024.100275 (PMC11617684; doi:10.1016/j.cpnec.2024.100275)
Supplement: Multimedia component 1 [file mmc1.docx]

**Supplementary Figure 1** – Flow diagram indicating the number of participants assessed, recruited, randomly assigned, and analysed. MMST = The Mannheim Multicomponent Stress Test; TSST = The Trier Social Stress Test.


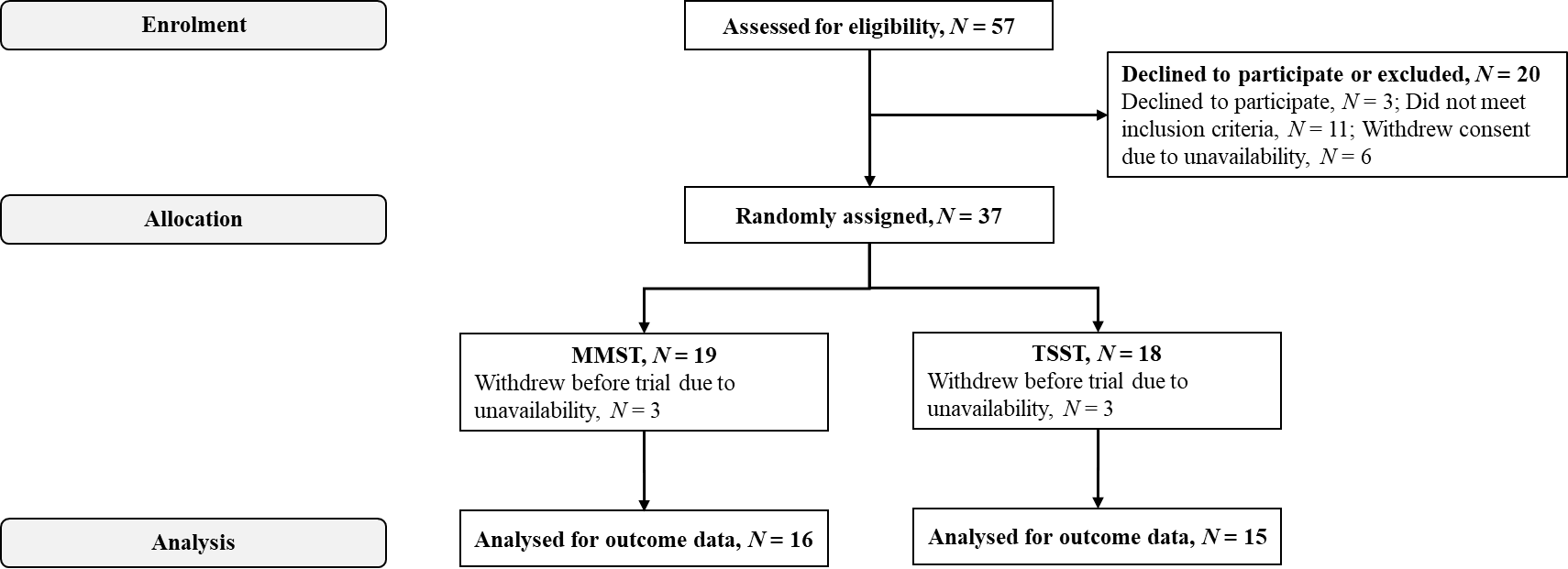


**Supplementary Figure 1** – Flow diagram indicating the number of participants assessed, recruited, randomly assigned, and analysed. MMST = The Mannheim Multicomponent Stress Test; TSST = The Trier Social Stress Test.

| **Supplementary table 1.** In-house pilot investigation demonstrating typical day-to-day biological variation for heart rate, saliva cortisol and saliva α-amylase. | | | | | | | | | | |
| --- | --- | --- | --- | --- | --- | --- | --- | --- | --- | --- |
|  |  | **Visit 1** |  | **Visit 2** |  | **Visit 3** |  | **M*_G_*** |  | **CV_I_ (%)** |
| Heart rate (bpm) |  | 72 ± 11 |  | 72 ± 11 |  | 71 ± 10 |  | 72 ± 10 |  | 5 |
| Saliva α-amylase (U/mL) |  | 131 ± 80 |  | 123 ± 92 |  | 137 ± 140 |  | 131 ± 92 |  | 25 |
| Saliva Cortisol (nmol/L) |  | 6 ± 6 |  | 6 ± 8 |  | 6 ± 8 |  | 6 ± 6 |  | 34 |
| In this pilot investigation, *n =* 24 healthy adults visited the laboratory in the afternoon on 3 occasions each separated by 1 week (12:00–15:30; time of day was matched for each participant). After a standardised 15-minute seated rest, baseline heart rate was recorded, and saliva samples were collected for subsequent analysis of α-amylase and cortisol. M*_G_ =* Grand mean, CV_I_ = Intra-individual day-to-day coefficient of variation. As the CV_I_ for heart rate was established while the participant was seated, yet the TSST requires the participant to stand during the free speech and mental arithmetic component, we used the CV_I_ for heart rate to identify meaningful heart rate responders to the MMST only. Values presented as mean ± SD unless otherwise stated. | | | | | | | | | | |
